# Myocarditis, pancreatitis, polyarthritis, mononeuritis multiplex and vasculitis with symmetrical peripheral gangrene of the lower extremities as a rare presentation of leptospirosis: a case report and review of the literature

**DOI:** 10.1186/1752-1947-8-150

**Published:** 2014-05-14

**Authors:** Periklis Panagopoulos, Irene Terzi, Michail Karanikas, Nikolaos Galanopoulos, Efstratios Maltezos

**Affiliations:** 1 2nd University Department of Internal Medicine, University General Hospital of Alexandroupolis, Democritus University of Thrace, Dragana, 68100 Alexandroupolis, Greece; 2 1st Department of Surgery, University General Hospital of Alexandroupolis, Democritus University of Thrace, Dragana, 68100 Alexandroupolis, Greece; 3 Department of Rheumatology, University General Hospital of Alexandroupolis, Democritus University of Thrace, Dragana Alexandroupolis, Greece

**Keywords:** Leptospirosis, Myocarditis, Vasculitis

## Abstract

**Introduction:**

Leptospirosis is a zoonosis caused by the spirochete, *Leptospira interrogans*. While most cases of leptospirosis are mild to moderate, the course may be complicated by multiorgan dysfunction. We present a rare case of leptospirosis with acute myocarditis, pancreatitis, polyarthritis, mononeuritis multiplex and severe vasculitis with necrosis of the extremities.

**Case presentation:**

A 32-year-old man from Congo presented with high-grade fever, confusion and headache. He developed tachycardia and hypotension followed by electrocardiogram changes and elevation of troponin I levels suggesting myocarditis. A physical examination revealed conjunctival suffusion, polyarthritis of his lower extremities and cutaneous necrosis of his feet due to vasculitis. Laboratory findings included amylase levels 10-fold the upper normal serum levels and thrombocytopenia. The diagnosis was confirmed by a positive leptospira immunoglobulin M, negative immunoglobulin G and a positive rapid agglutination test. Our patient recovered progressively with antimicrobials and supportive care.

**Conclusions:**

Because the clinical features and diagnostic findings of leptospirosis are not specific, a high index of suspicion must be maintained for the diagnosis. Serology is the most important tool for accurate and quick diagnosis in order to administer the appropriate therapy.

## Introduction

Leptospirosis is a zoonosis caused by the spirochete, *Leptospira interrogans*. The majority of the cases occur in the tropics, however, cases are also observed in temperate regions [1]. Leptospirosis may be complicated by multiorgan failure, such as renal failure, liver failure, acute respiratory distress syndrome and rhabdomyolysis [2]. Leptospirosis may be mistaken for diverse infectious diseases. Malaria, dengue fever, rickettsial disease, ehrlichiosis or hantavirus infection may mimic leptospirosis. We present a rare case of leptospirosis with acute myocarditis, pancreatitis and severe vasculitis with necrosis of the extremities.

## Case presentation

A 32-year-old man from Congo presented to another regional hospital with fever, headache, confusion, rigor and tachycardia, in addition to low arterial blood pressure (70/40mmHg) despite adequate fluid infusion. His fever and headache began a week before the first admission. An electrocardiogram revealed abnormalities (segment depression (ST)), whereas laboratory tests showed thrombocytopenia (platelets: 45000/mm^3^) and a more than 20-fold elevation of troponin I serum levels (>2000IU/ml). He presented with an unstable polymorphic ventricular tachycardia and was admitted to the Cardiology Intensive Care Unit, where he underwent defibrillation procedures, inotropic support and piperacillin plus tazobactam administration. After his stabilization he was transferred to the Infectious Diseases Unit of our hospital for further investigation.

On admission he was icteric, with conjunctival suffusion and arthritis in both knees and ankles. His blood pressure was 120/80mmHg without inotropic support, his oxygen saturation was 98% (FiO_2_: 21%), whereas the laboratory tests showed an elevated white blood cell (WBC) count (approximately 15000/mm^3^), thrombocytopenia (approximately 50000/mm^3^), amylase elevation (approximately 1000IU/ml), hypoglycemia, acute renal failure (creatinine levels: 3.0mg/dl), troponin I levels >2000 and elevated creatine kinase (CPK approximately 5000IU/ml) as well as aspartate and alanine aminotransferases (>x5).

Our patient had been in Greece for at least 15 months, he was previously healthy and the only risk factor for the specific infection was household exposure.

The transthoracic cardiac ultrasound revealed an ejection fraction lower than 30. A multiplex polymerase chain reaction (PCR) for *Neisseria meningitidis* was negative as well as the tests for Crimean-Congo hemorrhagic fever and hantavirus. A knee puncture was performed and depicted noninfectious arthritis (WBC <5000/mm^3^). Immunoglobulin M (IgM) antibodies for leptospira were positive (+), whereas the IgG antibodies were negative. In addition the rapid agglutination test for leptospira was positive (+). Urine, blood and synovial fluid samples were tested for leptospira with PCR, but all results were negative probably because the specimens were collected at least one week after the initiation of antibiotic therapy.

**Figure 1:**
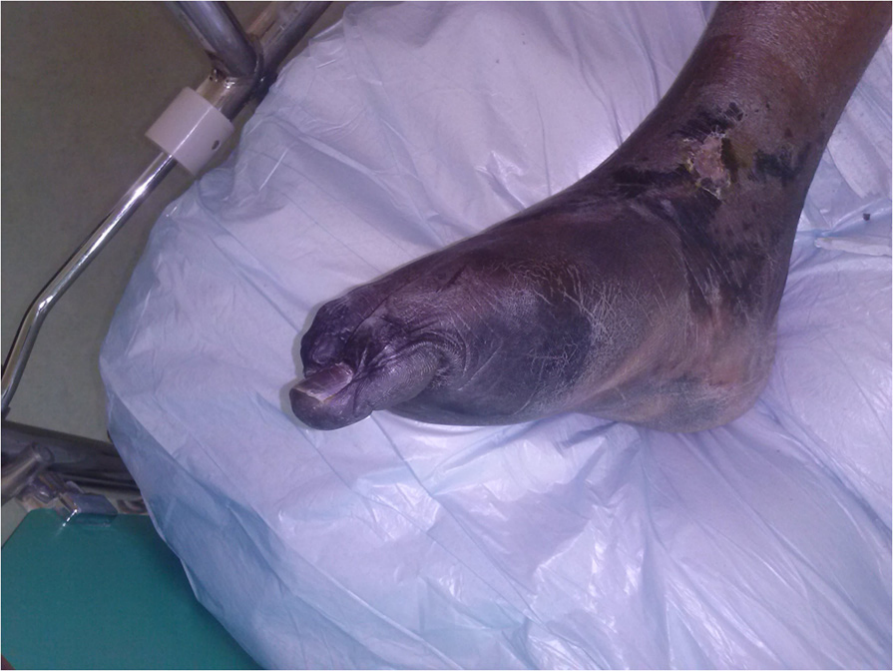
Skin necrosis of the lower extremity.

Penicillin (1,500,000 units every 6 hours) was administered (the drug of choice for leptospirosis), however, meropenem and vancomycin were also administered to our patient due to a probable nosocomial infection (severe sepsis). Cardiac and renal function were normalized within four weeks of hospitalization, whereas our patient became afebrile after the completion of Day 7 of therapy. Unfortunately, despite the rapid improvement of the myocarditis and of his general condition, a new complication occurred during the third week of hospitalization. A slow progressive necrosis of soft tissue in both feet due to vasculitis was diagnosed (Figure 1). X-rays and magnetic resonance imaging (MRI) were performed to exclude osteomyelitis. A conservative treatment was the option with antibiotics, vasodilators (ilomedin/iloprost), aspirin and minor surgical interventions instead of a both feet below the knee amputation.

At the same time, he presented with a second episode of polyarthritis, and mononeuritis multiplex, successfully controlled with prednisolone, pregabalin and analgesics.

Finally, 16 weeks after admission our patient was in excellent clinical condition, had progressively recovered and was free of further complications.

## Discussion

Leptospirosis is a disease with a variable grade of severity and can present either as a mild infection of a two-stage anicteric illness (80% of the cases) or as a severe icteric disease [3]. It is also known as Weil’s disease, Swineherd’s disease, Stuttgart disease, and Canicola fever. Humans most often become infected after exposure to environmental sources, such as animal urine, contaminated water or soil or infected animal tissue. Risk factors for infection include recreational activities (canoeing, kayaking, rafting, fresh water swimming), occupational exposure (sewer workers, military personnel, farmers) and household exposure [1,4]. All medical personnel should be aware of probable leptospirosis in order to avoid a delayed diagnosis due to the nonspecific clinical features. Many mild or severe complications can occur and are often immune-mediated, such as pulmonary hemorrhage, which has been related to Toll-like receptors (TLR) activation. Leptospira lipoprotein Lip32 triggers inflammatory responses in renal proximal tubule cells by activation of TLR2, nuclear factor (kappa) B and mitogen-activated protein kinases. Fortunately, there was no lung involvement in our case and the cornerstone sign for the diagnosis was conjunctival suffusion. Acute pancreatitis and myocarditis are very rare manifestations in leptospirosis [5]. In addition, vasculitis and gangrene are also uncommon complications [6]. The occurrence of such symptoms and signs several weeks after the subsidence of acute fever is strongly indicative of the second or immune phase of the disease. Of note, during the immune phase polyradiculoneuropathy may occur or there may be a combined involvement of muscle, nerve and myoneural junction [7,8]. Additionally, unusual or previously unreported manifestations of leptospirosis including infarction of the extremities in children or acute necrotizing retinitis could be explained by a similar autoimmune mechanism [9,10]. The laboratory gold standard test for the diagnosis is the microscopic agglutination test (MAT). Moreover, two other rapid, useful diagnostic tests, the microplate IgM enzyme-linked immunosorbent assay (ELISA) and IgM dot-ELISA dipstick tests, are available.

The mean incubation period is 10 days (range 2 to 30 days), although determination of precise exposures may be difficult [11]. Our patient described a sudden onset of symptoms although the mode of exposure was unclear.

## Conclusions

Leptospirosis is a disease commonly underdiagnosed in humans because is often mild, asymptomatic or presents with nonspecific symptoms. The broad spectrum of severity as well as the unusual or rare clinical manifestations may result in a delay in the diagnosis. There is a need to increase awareness of the disease so that timely therapy can be instituted to patients.

Because the clinical features and diagnostic findings of leptospirosis are not specific a high index of suspicion is necessary for the diagnosis. Serology is the most important tool for an accurate and quick diagnosis in order to administer the appropriate therapy.

## Consent

Written informed consent was obtained from the patient for publication of this case report and any accompanying images. A copy of the written consent is available for review by the Editor-in-Chief of this journal.

## Abbreviations

CPK: creatine kinase; IgG/ M: immunoglobulin G/M; IU: international units; MAT: microscopic agglutination test; MRI: magnetic resonance imaging; PCR: polymerase chain reaction; TLR: Toll-like receptors; WBC: white blood cell.

## Competing interests

The authors declare that they have no competing interests.

## Authors’ contributions

All authors contributed equally in writing the manuscript. All authors read and approved the final manuscript.
